# Impaired sleep, depressive symptoms, and pain as determinants of physical activity and exercise intervention adherence: an exploratory analysis of a randomized clinical trial

**DOI:** 10.1186/s12877-025-05830-y

**Published:** 2025-03-29

**Authors:** Eija Kekkonen, Anette Hall, Riitta Antikainen, Satu Havulinna, Miia Kivipelto, Jenni Kulmala, Tiina Laatikainen, Teemu I. Paajanen, Shireen Sindi, Hilkka Soininen, Timo Strandberg, Jaakko Tuomilehto, Tiia Ngandu, Alina Solomon

**Affiliations:** 1 Department of Neurology, Institute of Clinical Medicine, University of Eastern Finland, P.O. Box 1627, Kuopio, FI-70211 Finland; 2 Division of Clinical Geriatrics, Center for Alzheimer Research, Department of Neurobiology, Care Sciences and Society, Karolinska Institute, Stockholm, Sweden; 3 Center for Life Course Health Research/Geriatrics, University of Oulu, Oulu, Finland; 4 Department Public Health, Finnish Institute for Health and Welfare, Helsinki, Finland; 5 Ageing Epidemiology Research Unit, School of Public Health, Imperial College London, London, UK; 6 Institute of Public Health and Clinical Nutrition, University of Eastern Finland, Kuopio, Finland; 7 Faculty of Social Sciences (Health Sciences) and Gerontology Research Center (GEREC), Tampere University, Tampere, Finland; 8 Work Ability and Working Careers, Finnish Institute of Occupational Health, Helsinki, Finland; 9 Department of Medicine, Geriatric Clinic, University of Helsinki and Helsinki University Central Hospital, Helsinki, Finland; 10 Department of Public Health, University of Helsinki, Helsinki, Finland

**Keywords:** Physical activity, Exercise, Sleep, Mood, Pain, Lifestyle intervention, Adherence

## Abstract

**Background:**

Physical activity (PA) and exercise interventions offer health benefits can reduce dementia risk. However, there might be barriers to engage in PA, such as sleep problems, depressive symptoms and pain, which are common complaints with older adults. We investigated sleep duration, sleep quality, depressive symptoms, and pain at baseline as potential determinants of: (i) adherence to the exercise intervention component of a 2-year multidomain lifestyle intervention; (ii) intervention’s effect on PA after 2 years; and (iii) overall PA after 2 years (exploratory analyses).

**Methods:**

The FINGER trial included 1259 individuals at risk for dementia, aged 60–77 years who were randomized (1:1) to a multidomain lifestyle intervention (exercise, diet, cognitive training, vascular risk factor management) or a control (regular health advice) group. Logistic regression analyses were used with exercise adherence (adherent: ≥66% participation) or self-reported PA (active: ≥2 times/week) as outcomes, adjusted for relevant baseline characteristics. Data on PA at baseline and at 2-years were available for 1100 participants.

**Results:**

Adherence to the exercise intervention was less likely with sleep duration < 6 h or ≥ 9 h per night compared with 7–8 h. OR (95% CI) were 0.46 (0.21–0.99) and 0.38 (0.20–0.74), respectively. The intervention group was more likely to be physically active than the control group at two years (OR 1.87, 95% CI 1.36–2.55). This intervention benefit did not significantly vary by baseline sleep duration, depressive symptoms, or pain (*p* > 0.3 for all interactions). Regardless of randomization group, those sleeping < 6 h were less likely to be physically active at two years, compared with participants sleeping 7–8 h (OR 0.36, 95% CI 0.18–0.72). Depressive symptoms or pain were not related to PA at two years.

**Conclusions:**

Older adults with sleep problems, depressive symptoms, or pain may benefit from lifestyle interventions. However, both short and long sleep duration can pose barriers to engaging in exercise intervention and should be carefully considered when designing strategies to promote PA among older populations at risk for dementia.

**Trial registration:**

The FINGER trial was registered at ClinicalTrials.gov with identifier NCT01041989 on 04/01/2010.

**Supplementary Information:**

The online version contains supplementary material available at 10.1186/s12877-025-05830-y.

## Introduction

Older adults meeting physical activity (PA) recommendations have a reduced risk of non-communicable diseases such as cardiovascular disease [1, 2] and diabetes [3]. Sufficient PA has been associated with reduced risk of cognitive decline and dementia, particularly Alzheimer’s disease (AD) [4, 5]. Older adults are the most sedentary age group [6]. Furthermore, as ageing is associated with a higher risk for cognitive decline and other health-related challenges [5, 7], it is crucial to investigate potential barriers to participation in lifestyle intervention programs including maintaining a physically active lifestyle. Sleep problems, depressive symptoms, and pain are common complaints in late life [8–11], often co-occurring [12–14], impacting negatively on PA. This impact on PA has been less investigated in the context of intervention studies and most earlier studies have focused primarily on how PA affects sleep, depressive symptoms and pain [15, 16].

Two observational studies showed the previous night actigraphy measured sleep efficiency and self-reported longer sleep duration predicting greater PA level the next day in older adults [17, 18]. Similarly, a 2-year longitudinal observational study in community-dwelling older adults reported that prior self-reported poor sleep quality predicted lower PA [19]. An 18-week lifestyle intervention study showed that better self-reported sleep quality was associated with increased PA the next day [20]. Depressive symptoms have been associated with physical inactivity in older populations [21, 22]. For instance, in a longitudinal study, depressive symptoms predicted lower levels of PA after two years [23]. A few lifestyle intervention studies have reported lower exercise participation rates in people with increased depressive symptoms [24–27]. Pain, often resulting from age-related musculoskeletal conditions or other causes, has also been associated with lower levels of PA [28–30]. However, the associations of impaired sleep or pain on exercise intervention participation have been poorly explored previously, particularly in the context of dementia prevention.

Multidomain lifestyle interventions including exercise are increasingly recognised as a promising dementia risk reduction strategy [5, 31]. The Finnish Geriatric Intervention Study to Prevent Cognitive Impairment and Disability (FINGER) was the first large-scale long-term randomized controlled trial to show that a 2-year multidomain lifestyle intervention - combining exercise, diet, cognitive training and vascular risk factor management could improve or maintain cognition in older adults at risk for dementia [31]. Cognitive benefits were also associated with better adherence to the intervention [32]. This study investigated self-reported sleep duration, sleep quality, depressive symptoms, and bodily pain at baseline as potential determinants of: (i) adherence to the exercise component within the intervention group; (ii) intervention effect on PA after two years; and (iii) overall PA levels after two years in all trial participants (exploratory analyses).

## Methods

This study is reported in accordance with the Consolidated Standards of Reporting Trials (CONSORT) guidelines.

### Population and intervention

FINGER (ClinicalTrials.gov identifier NCT01041989) was a 2-year randomized controlled trial conducted at six clinical sites in Finland involving 1259 older individuals at risk for dementia. The detailed protocol and primary results have been previously published [33]. Participants were aged 60–77 years, had a Cardiovascular Risk Factors, Aging and Dementia (CAIDE) risk score of ≥ 6 points - indicating higher dementia risk, and cognitive performance around the mean level or slightly lower than expected for age according to Finnish population norms [31]. Participants with dementia or conditions affecting safe participation were excluded. Eligible participants were randomized (1:1) into multidomain intervention and control groups, in blocks of four (two individuals randomly allocated to each group) at each site after baseline by the study nurse. Double blinding was implemented as much as possible: outcome assessors were blinded, and participants were not explicitly informed about their randomization group.

The control group received general health advice. The intervention group received a multidomain intervention comprising several components: physical exercise, nutritional guidance, cognitive training, social stimulation, and monitoring and management of metabolic and vascular risk factors. The exercise component followed international guidelines and included group training sessions at the gym, guided by the study physiotherapists. The training program was individually tailored based on repetition maximum measurements and incorporated progressive muscle strength training (1–3 times/week) and aerobic exercise (2–5 times/week), including exercises to improve postural balance [31, 33].

The primary outcome of the FINGER trial was change in cognition measured with a comprehensive neuropsychological test battery (NTB) total score [31]. The present study represents an exploratory analysis investigating baseline sleep, depressive symptoms, and pain as potential determinants of exercise adherence and self-reported physical activity at the 2-year follow-up.

### Assessment of physical activity, sleep, depressive symptoms, and pain

Exercise adherence in the intervention group was defined as the percentage of participation in the offered gym sessions. Participants were considered adherent if they attended in at least 66% of the exercise training sessions, a previously proposed suitable cut-off for behavioral interventions [32, 34].

PA in all trial participants was assessed as the self-reported frequency of exercise activities lasting at least 20 min and having sufficient intensity to cause slight breathlessness and sweating. Based on a previously defined cut-off, participants reporting a frequency of at least two times per week were considered physically active [35].

Self-reported average duration of sleep during the night was categorized as < 6 h, ≥ 6–<7 h, ≥ 7–<8 h, ≥ 8–<9 h, and ≥ 9 h. The recommended sleep duration for older adults is ≥ 7–<8 h per night [36]. Sleep quality was calculated as a composite index based on seven sleep-related questions (Appendix Table 3). A cut-off of ≥ 40/100 was used to define poor sleep quality. Depressive symptoms were assessed using the Zung scale, and cut-off for at least mild depressive symptoms was set to ≥ 40, as previously defined [37–39]. Pain was measured using a composite index based on the RAND-36 bodily pain scale [40]; cut-off was set to ≥ 40/100 for indicating at least moderate pain that interferes with daily activities and work (Appendix Table 3).

### Statistical analysis

**Fig. 1:**
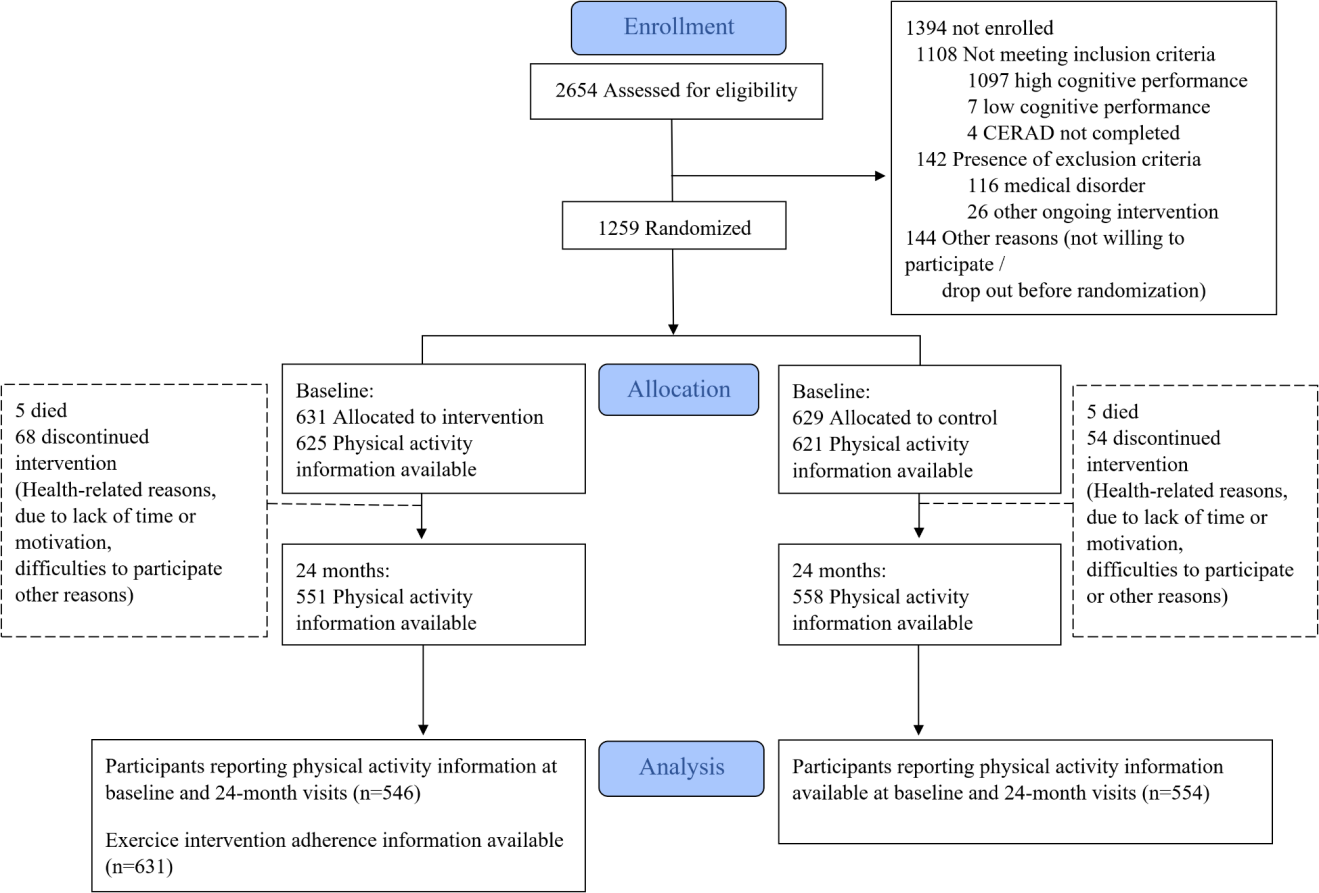
Study flowchart

Data analyses in this study were exploratory. A study flowchart illustrating the study design with a focus on PA and exercise intervention adherence as the outcomes is presented in Fig. 1.

#### Descriptive analyses

Between-group differences in baseline characteristics were analyzed with t-test and one-way ANOVA for normally distributed variables, Mann-Whitney test or Kruskal-Wallis test for non-normally distributed variables, and Chi-square test for categorical variables. Cross-sectional associations between sleep duration, sleep quality, depressive symptoms or pain and PA at baseline (active versus inactive) were analyzed using logistic regression models, adjusted for study site and other potential confounders (age, sex, education, body mass index (BMI), marital status, self-rated health status).

#### Baseline sleep, depressive symptoms and pain as potential determinants of exercise intervention adherence (adherent versus non-adherent, intervention group participants)

Logistic regression models were adjusted for study site, baseline PA, age, sex, education, BMI, marital status, self-rated health status (Model 1). Model 2 additionally adjusted for the potential impact of relevant baseline medications (for sleep problems, depressive symptoms or pain). Model 3 was based on Model 1, with additional adjustment for the potential impact of baseline cognitive performance (total NTB score). For sleep, the recommended duration of 7–8 h/night was used as the reference category [36]. The effects of depressive symptoms on exercise adherence have been reported previously [24, 27].

#### Baseline sleep, depressive symptoms, and pain as potential determinants of intervention effect on self-reported PA (active/inactive) after 2 years

Analyses included all intervention and control group participants with PA data available from both baseline and 2-year visits. To assess the intervention effect on PA, logistic regression was conducted with PA after the 2-year trial as dependent variable and randomization group as independent variable, adjusted as in Model 1 above. To evaluate whether the effect on PA was modified by baseline sleep duration or quality, depressive symptoms or pain, the following terms were separately added to the logistic regression model: (i) sleep duration and sleep duration×randomization group interaction; (ii) sleep quality and sleep quality×randomization group interaction; (iii) depressive symptoms and depressive symptoms×randomization group interaction; and (iv) pain and pain×randomization group interaction. We report the two-way interactions and the intervention effect stratified by sleep duration, sleep quality, depressive symptoms, and pain categories.

#### Baseline sleep, depressive symptoms, and pain as potential determinants of overall PA after 2 years

Logistic regression models were conducted with PA at the 2-year follow-up as the dependent variable, and sleep, depressive symptoms, or pain as independent variables, adjusted as in Models 1–3 above.

All analyses were conducted using STATA version 14 software, and the significance level was set to 0.05.

## Results

A total of 1100 participants (87.4%) had PA data available at both baseline and 2-year visits (Fig. 1). At baseline, there were no significant differences between intervention and control groups. Overall, compared with participants who were active at baseline, inactive participants had a higher BMI, worse self-rated health, poorer sleep quality, more pain and more depressive symptoms (Table 1). Participants without available PA data (*n* = 159) were slightly older, had a lower education and cognition, and more pain, with a trend toward more frequently short or long sleep duration than participants with available PA data (Appendix Table 4). Sleep duration, sleep quality, depressive symptoms and pain were all significantly associated with each other at baseline (Appendix Table 5, and 6). Cross-sectional analyses at baseline showed that participants with depressive symptoms or shorter sleep duration (≥ 6–<7 h compared with ≥ 7–<8 h/night) were less likely to be physically active: (OR 0.70, 95% CI 0.50–1.00) and (OR 0.66, 95% CI 0.44–1.00), respectively.

**Table 1:** Baseline characteristics of the FINGER participants with available physical activity data at both baseline and the 2-year follow-up

	Intervention Group *N* = 546	Control Group *N* = 554	*p*-value	Physically active ≥ 2 times a week *N* = 786	Physically active < 2 times a week *N* = 314	*p* value
Age, mean (SD)	68.9 (4.6)	68.5 (4.7)	0.172	68.8 (4.7)	68.5 (4.59)	0.301
Sex, female n (%)	242 (44.3%)	265 (47.8%)	0.243	371 (47.2%)	136 (43.3%)	0.243
Education years, mean (SD)	10.0 (3.5)	10.1 (3.5)	0.816	10.0 (3.4)	10.1 (3.5)	0.986
Body mass index, mean (SD)	28.2 (4.5)	28.0 (5.0)	0.546	27.8 (4.4)	29.2 (5.4)	< 0.001
Self-rated health						
Poor self-rated health, n (%)	18 (3.3%)	18 (3.3%)	0.492	6 (0.8%)	30 (9.6%)	< 0.001
Average self-rated health, n (%)	204 (37.6%)	189 (34.2%)		257 (32.9%)	136 (43.3%)	
Excellent or good self-rated health, n (%)	321 (59.1%)	346 (62.6%)		519 (66.4%)	148 (47.1%)	
Cognition (NTB total score), mean (SD)	-0.02 (0.56)	0.05 (0.58)	0.039	0.01 (0.57)	0.02 (0.59)	0.845
Physical activity at least 2 times a week, n (%)	383 (70.2%)	403 (72.7%)	0.340			
Average sleep duration during the night						
Sleep duration of < 6 h	33 (6.0%)	21 (3.8%)	0.532	39 (5.0%)	15 (4.8%)	0.084
Sleep duration of 6–7 h	85 (15.6%)	88 (16.0%)		111 (14.2%)	62 (19.8%)	
Sleep duration of 7–8 h	190 (34.9%)	197 (35.7%)		292 (37.2%)	95 (30.4%)	
Sleep duration of 8–9 h	188 (34.5%)	199 (36.0%)		277 (35.3%)	110 (35.1%)	
Sleep duration of ≥ 9 h	49 (9.0%)	47 (8.5%)		65 (8.3%)	31 (9.9%)	
Sleep quality index, mean (SD)	25.2 (17.0)	26.0 (17.2)	0.452	24.3 (15.7)	38.8 (19.6)	< 0.001
Depressive symptoms, mean (SD)	34.0 (7.7)	33.8 (7.1)	0.614	33.2 (7.1)	35.6 (7.9)	< 0.001
Bodily pain, mean (SD)	25.3 (22.2)	25.0 (21.3)	0.796	23.6 (20.3)	29.1 (24.7)	0.002
*At least 66% exercise intervention adherence (intervention group only), n (%)	304 (48.2%)			233 (53.4%)	69 (36.5%)	< 0.001

NTB = Neuropsychological test battery

Numbers are means (SD), unless otherwise specified. P-values are shown from t-test for normally distributed variables and Mann-Whitney test for non-normally distributed variables (Sleep quality and Bodily pain), and chi square test for categorical variables. * Exercise adherence in the intervention group was defined as the percentage of participation in the offered gym sessions. Participants were considered adherent if they attended at least 66% of the exercise training sessions. The table shows number (%) of adherent participants

### Baseline sleep, depressive symptoms and pain as potential determinants of exercise intervention adherence

Of the intervention group participants, 48% adhered to the exercise training program. Adherence varied by sleep duration. The lowest adherence rates (32% and 33%) were observed among participants with the longest (≥ 9 h) and shortest (< 6 h) sleep duration. Participants sleeping ≥ 6–<7 h, ≥ 7–<8 h, ≥ 8–<9 h were more often adherent (51%, 52% and 50%, respectively). As expected, adherent participants were also more likely to be physically active after the 2-year intervention (OR 3.37, 95% CI 1.99–5.73).

Participants sleeping either < 6 h or ≥ 9 h were significantly less likely to be adherent compared with participants sleeping ≥ 7–<8 h, (OR 0.46, 95% CI 0.21–0.99) and (OR 0.38, 95% CI 0.20–0.74), respectively (Model 1, Table 2). Restricting the analysis to participants with PA data available at both baseline and 2-year visits did not substantially change the results. Further adjustment for sleep medication (Model 2) or baseline cognition (Model 3) resulted in consistent findings. The effect of baseline short (< 6 h) and long (≥ 9 h) sleep duration on exercise intervention adherence remained similar in additional analyses adjusting Model 1 for depressive symptoms and pain (OR 0.37, 95% CI 0.16–0.88), and (OR 0.39, 95% CI 0.19–0.80), respectively. Sleep quality and pain were not significantly associated with exercise intervention adherence (Table 2).

**Table 2:** The impact of baseline sleep duration and quality, and pain on exercise adherence in the intervention group

	Model 1. Including all intervention group participants		Model 2. Including all intervention group participants	Model 3. Including all intervention group participants	Intervention group participants with PA information available	
	N	OR (95% CI)	OR (95% CI)	OR (95% CI)	N	OR (95% CI)
Sleep duration < 6 h	614	**0.46** **(0.21–0.99)**	0.41 (0.21–1.01)	0.46 (0.21–1.01)	536	**0.43** **(0.19–0.97)**
Sleep duration ≥ 6–<7 h		1.18 (0.70–2.01)	1.20 (0.71–2.05)	1.19 (0.70–2.02)		1.13 (0.64–2.02)
Sleep duration ≤ 7–<8 h (ref)		1	1	1		1
Sleep duration ≥ 8–<9 h		0.85 (0.56–1.29)	0.84 (0.55–1.27)	0.85 (0.56–1.29)		0.79 (0.50–1.24)
Sleep duration ≥ 9 h		**0.38** **(0.20–0.74)**	**0.37** **(1.9–0.72)**	**0.39** **(0.20–0.75)**		**0.40** **(0.20–0.81)**
Better sleep quality (index < 40)	615	1	1	1	536	1
Worse sleep quality (index ≥ 40)		1.2 (0.78–1.90)	1.29 (0.81–2.05)	1.22 (0.78–1.91)		1.1 (0.68–1.78)
No or mild bodily pain (RAND-36 Bodily pain < 40)	609	1	1	1	533	1
Pronounced bodily pain (RAND-36 Bodily pain ≥ 40)		0.90 (0.59–1.37)	0.80 (0.51–1.24)	0.90 (0.59–1.39)		0.87 (0.55–1.37)

ORs (95% CI) are shown from binary logistic regression models with at least 66% exercise adherence to the FINGER multidomain intervention as the outcome. Results are presented for all intervention participants, as well as separately for those who reported physical activity data at both baseline and at the 2-year follow-up. Values in bold correspond to *p* < 0.05

Model 1Adjusted for baseline physical activity, age, sex, education years, BMI, marital status and self-reported current health status at baseline

Model 2 is adjusted as model 1 and additionally adjusted for medications

Model 3 is adjusted as model 1 and additionally adjusted for cognition

Results for depressive symptoms were reported previously (27)

### Baseline sleep, depressive symptoms, and pain as potential determinants of intervention effect on PA at the 2-year follow-up

As expected, participants who were physically active at baseline were more likely to remain physically active also after 2 years, regardless of the randomization group (OR 7.60, 95% CI 5.51–10.48). PA increased significantly more in the intervention group compared with control group (OR 1.87, 95% CI 1.36–2.55). This intervention benefit on self-reported PA did not significantly differ by baseline sleep duration or quality, depressive symptoms, or pain (*p* > 0.3 for all interactions, Table 3). In stratified analyses by baseline sleep duration, sleep quality, depressive symptoms, or pain (Table 3), all ORs for intervention-control differences in 2-year PA indicated a favorable intervention effect on PA regardless of these baseline characteristics (ORs from 1.68 to 5.46), except for long sleep duration (OR = 1).

**Table 3:** Intervention effect on PA after 2 years, stratified by baseline sleep duration and quality, depressive symptoms, and pain

		Likelihood of being physically active after 2 years in the intervention compared with control group		Difference between sleep, depressive symptoms or pain groups
	N	OR	95% CI	p-value, interaction with randomization group
Sleep duration < 6 h	50	5.46	0.79–37.55	0.711
Sleep duration ≥ 6–<7 h	169	1.68	0.77–3.66	0.779
Sleep duration ≤ 7–<8 h (ref)	379	**2.15**	**1.24–3.75**	REF
Sleep duration ≥ 8–<9 h	376	**2.05**	**1.16–3.63**	0.738
Sleep duration ≥ 9 h	96	1.00	0.31–3.27	0.322
Better sleep quality (index < 40)	874	**1.89**	**1.33–2.70**	0.921
Worse sleep quality (index ≥ 40)	205	1.95	0.98–3.88	
No depressive symptoms (Zung score < 40)	842	**1.74**	**1.21–2.49**	0.302
At least mild depressive symptoms (Zung score ≥ 40)	217	**2.75**	**1.32–5.69**	
No or mild bodily pain (RAND-36 Bodily pain < 40)	794	**1.70**	**1.18–2.46**	0.421
Pronounced bodily pain (RAND-36 Bodily pain ≥ 40)	280	**2.62**	**1.35–5.08**	

ORs (95% CI) are presented from logistic regression models with physical activity after 2 years as the outcome, and adjusted for randomization group, baseline physical activity, age, sex, years of education, BMI, marital status and self-reported current health status at baseline. Models are stratified by baseline sleep duration, sleep quality, depressive symptoms, or pain. Values in bold indicate *p* < 0.05 for ORs within each sleep, depressive symptoms, or pain group. P-values for interactions of sleep, depressive symptoms, or pain groups and randomization group indicated no statistically significant differences in intervention effect on PA across these groups

### Baseline sleep, depressive symptoms, and pain as potential determinants of overall PA after 2 years

Compared with a sleep duration of ≥ 7–<8 h sleep, participants who slept < 6 h at baseline were less likely to be physically active at two years, (OR 0.36, 95% CI 0.18–0.72) (Model 1, Table 4). Results were consistent after additional adjustments for sleep medication (Model 2) and baseline cognition (Model 3). Further adjustment in Model 1 for depressive symptoms and pain did not alter the results (OR 0.34, 95% CI 0.17–0.67). A similar trend was observed for participants sleeping ≥ 6–<7 h compared with ≥ 7–<8 h: (OR 0.65, 95% CI 0.41–1.03); and for poor versus better sleep quality (OR 0.68, 95% CI 0.46 − 1.01) (Table 4). Other sleep duration categories, depressive symptoms, or pain were not significantly associated with PA after 2 years.

**Table 4:** Overall impact of baseline sleep duration and quality, depressive symptoms, and pain on physical activity after 2 years (intervention and control participants combined, including participants with physical activity information available at baseline and at 2-years)

		Model 1.	Model 2.	Model 3.
	N	**OR (95% CI)**	**OR (95% CI)**	**OR (95% CI)**
Sleep duration < 6 h	1079	**0.36** **(0.18–0.72)**	**0.36** **(0.18–0.73)**	**0.39** **(0.19–0.78)**
Sleep duration ≥ 6–<7 h		0.65 (0.41–1.03)	0.65 (0.41–1.03)	0.65 (0.41–1.03)
Sleep duration ≤ 7–<8 h (ref)		1	1	1
Sleep duration ≥ 8–<9 h		1.00 (0.68–1.46)	0.98 (0.67–1.44)	1.01 (0.69–1.47)
Sleep duration ≥ 9 h		0.89 (0.50–1.59)	0.88 (0.49–1.57)	0.89 (0.50–1.59)
Better sleep quality (index < 40)	1079	1	1	1
Worse sleep quality (index ≥ 40)		0.68 (0.46–1.01)	0.71 (0.47–1.06)	0.70 (0.47–1.03)
No depressive symptoms (Zung score < 40)	1059	1	1	1
At least mild depressive symptoms (Zung score ≥ 40)		1.06 (0.71–1.58)	1.09 (0.73–1.62)	1.04 (0.70–1.57)
No or mild bodily pain (RAND-36 Bodily pain < 40)	1074	1	1	1
Pronounced bodily pain (RAND-36 Bodily pain ≥ 40)		0.90 (0.62–1.30)	0.86 (0.59–1.25)	0.90 (0.61–1.29)

ORs (95% CI) are shown from binary logistic regression models, with physical activity after 2 years as the outcome. Values in bold indicate *p* < 0.05. Model 1 is adjusted for randomization group, baseline physical activity, age, sex, years of education, BMI, marital status, and self-reported current health status at baseline. Model 2 includesthe adjustments from Model 1, with additional adjustments for medications. Model 3 includes adjustments from Model 1, with further adjustment for cognition

## Discussion

In the context of multidomain lifestyle interventions for dementia risk reduction, this study is among the first to investigate the associations of sleep, depressive symptoms, and pain with both the exercise intervention adherence and PA after a 2-year trial targeting older adults at risk for dementia. Exercise intervention adherence was lower in participants with both the shortest (< 6 h) and longest (≥ 9 h) sleep duration, and in those with depressive symptoms, as previously reported [27]. However, pain did not affect exercise adherence in this study. Self-reported PA after 2 years improved significantly more in the intervention group compared with the control group. This intervention benefit on PA was not significantly influenced by baseline sleep duration or quality, depressive symptoms, or pain levels. This is particularly important since participants with < 6 h/night sleep duration were overall less likely to be physically active after 2 years in both randomization groups, and independently of depressive symptoms and pain. A similar trend was also observed for participants with ≥ 6–<7 h sleep duration (compared with the recommended ≥ 7–<8 h) and those with poor sleep quality. Overall, depressive symptoms or pain were not significantly associated with PA after 2 years. The FINGER intervention included a structured and intensive group exercise program at the gym, supervised by a physiotherapist, which may have enhanced motivation and participation, thereby facilitating the intervention’s positive effect on PA.

A large body of previous research has focused on the beneficial effects of PA on various aspects of sleep [41, 42], particularly in the context of exercise programs [16]. However, the role of sleep as a determinant of both exercise intervention adherence and overall PA level has not been previously investigated in large-scale, longer-term randomized controlled trials. Previous observational studies have highlighted associations between sleep duration and PA. For instance, in a cross-sectional study of older adults, participants reporting short or long sleep, compared with the recommended sleep duration, were less likely to engage in PA [43]. In a prospective study of older adults, longer or shorter sleep duration (more or less than 6–8 h per night) was associated with a lower hand-grip strength and reduced PA levels [44]. Additionally, prior sleep quality was found to predict future PA levels, but not vice versa, in a 2-year follow-up study of community-dwelling older adults [19]. In a short-term (18-week) lifestyle intervention study, sleep quality emerged as a predictor of PA in older adults [20].

The relationship between PA and sleep is complex, particularly in the context of dementia prevention, as the study population of the present research was at increased risk of dementia. A sufficient PA level has been shown to reduce the risk of cardiovascular disease [1, 2] and diabetes [3], while both short and long sleep durations have been associated with an increased risk for coronary artery disease, stroke, diabetes [45, 46], and hypertension [46, 47], all of which are associated with dementia [5].Short sleep duration (≤ 6 h/night) in mid-life has been associated with an increased higher incidence of dementia in a > 30-year follow-up study, but in later life long sleep duration has also been associated with dementia [48]. The relationship between sleep and dementia is bidirectional as the development of AD-related pathology can disrupt sleep structure, and vice versa [49]. In the present study among older adults without substantial cognitive impairment but at risk for dementia, baseline sleep duration did not modify exercise intervention adherence and PA after two years, even adjusting for baseline cognition. These findings suggest that older adults at risk of dementia who have difficulties engaging in PA and experience either short or long sleep durations may benefit from support in both exercise and sleep management. While the exact mechanisms remain unclear, it has been suggested that daytime fatigue due to insufficient sleep might play a role in the association between sleep and PA [17]. Even though exercise is a well-established non-pharmacological intervention for improving sleep quality, its impact on short or long sleep duration remains less studied [50]. Future research should explore whether sleep problems would be associated with AD-related biomarkers or pathology in people at risk of dementia, and whether our findings would also apply to individuals with mild cognitive impairment.

Depressive symptoms have been associated with poorer adherence to several intervention domains including exercise in multidomain lifestyle interventions [24, 32]. However, in this study, participants with depressive symptoms still demonstrated an intervention benefit on PA after two years. Notably, participants had relatively low levels of depressive symptoms, which might have influenced the results. Although pain has been previously associated with lower levels of PA [28, 51], it did not significantly impact adherence to exercise intervention or PA at the 2-year follow-up. Participants reporting pain at baseline appeared to benefit from the FINGER intervention, potentially due to the flexibility for individual adjustments embedded within the standardized exercise training protocol.

A key strength of the present study lies in its the large-scale, long-term randomized controlled trial design. The trial targeted older adults with risk factors for dementia, including cardiovascular risk factors, making the identification of determinants for maintaining a physically active lifestyle particularly important. Furthermore, the exercise training program combined progressive muscle strength training and aerobic exercise guided by physiotherapists and tailored for individual needs. The adherence rate was meticulously tracked during the entire trial, providing robust insights into the intervention’s implementation and outcomes.

The study has several limitations. PA and sleep measures were self-reported and sleep quality was not assessed with a validated questionnaire or objective sleep registering. Additionally, data on other important aspects of sleep, such as depth and regularity, which have gained increasing attention in recent research [52, 53], were not available. The FINGER trial eligibility criteria excluded participants with substantial cognitive impairment, ongoing severe major depression or health conditions that could hinder safe engagement in the intervention. While these criteria ensured participants’ safety, they may limit the generalizability of the findings. Furthermore, participants with missing PA data were older, had lower education and cognition, and reported more pain and more frequent short or long sleep durations, which may have affected the results.

## Conclusions

In conclusion, older adults at risk of dementia who experience challenges in maintaining PA due to sleep problems or depressive symptoms may still benefit from lifestyle interventions that include exercise. Pain did not seem to affect exercise adherence or PA. However, given that inadequate sleep duration is associated with depressive symptoms and numerous other health issues, particular attention should be given to address short and long sleep duration and depressive symptoms within exercise interventions targeting people at risk of dementia. Future trials should explore whether managing sleep problems and depressive symptoms in this population can further enhance their ability to remain physically active. Additionally, multidomain lifestyle intervention trials should comprehensively examine the effects of sleep, depressive symptoms, and pain across all intervention components to maximize their potential benefits.

## Electronic supplementary material

Below is the link to the electronic supplementary material.

Supplementary Material 1

## Data Availability

Data used in this study is not publicly available due to ethical and legal reasons, but the data are available upon request. Those fulfilling the requirements for viewing confidential data as required by the Finnish legislation and the Finnish Institute for Health and Welfare are able to access the data after completion of a material transfer agreement. Requests may be directed to kirjaamo@thl.fi.

## Abbreviations

AD: Alzheimer’s disease
BMI: Body mass index
CAIDE: Cardiovascular Risk Factors, Aging and Dementia
FINGER: The Finnish Geriatric Intervention Study to Prevent Cognitive Impairment and Disability
NTB: Neuropsychological test battery
PA: Physical activity
RAND-36: The RAND 36-Item Health Survey 1.0
