# Supplementary material for: Impaired sleep, depressive symptoms, and pain as determinants of physical activity and exercise intervention adherence: an exploratory analysis of a randomized clinical trial
Source: BMC Geriatr. 2025 Mar 29;25:211. doi: 10.1186/s12877-025-05830-y (PMC11954347; doi:10.1186/s12877-025-05830-y)
Supplement: Supplementary file 1 — Supplementary Material 1 [file 12877_2025_5830_MOESM1_ESM.docx]

**Additional file**

**Association of impaired sleep, depressive symptoms and pain with physical activity and exercise intervention adherence**

**Table of contents:**

Appendix Table 1. Detailed inclusion and exclusion criteria in the FINGER trial.

Appendix Table 2. Cardiovascular Risk Factors, Aging, and Dementia (CAIDE) risk score.

Appendix Text 1. Description of the intervention procedures.

Appendix Table 3. Description of sleep, pain and depressive symptoms variables in detail.

Appendix Text 2. Cross-sectional results.

Appendix Table 4. Baseline characteristics of FINGER participants with no physical activity information from baseline and 24month visit compared to participants with available physical activity information at both timepoints.

Appendix Table 5. Baseline characteristics of FINGER participants according to sleep duration.

Appendix Table 6. Baseline characteristics by sleep quality, pain and depressive symptoms

References

## **Appendix Table 1. Detailed inclusion and exclusion criteria in the FINGER trial.**

|  | **Inclusion criteria** | **Exclusion criteria** |
| --- | --- | --- |
| Age | 60-77 years |  |
| Non-communicable diseases, conditions and lifestyle | Cardiovascular Risk Factors, Aging, and Dementia (CAIDE) risk score of ≥6 points (range 0-15 points)* | Conditions affecting safe engagement in the intervention (especially the exercise component), malignant diseases, major depression, symptomatic cardiovascular disease, revascularization within 1-year, severe loss of vision, hearing, or communicative ability |
| Cognition | Cognitively normal tested with Consortium to Establish a Registry for Alzheimer Disease (CERAD) neuropsychological battery.  One of the following:   - Word list learning task (10 words repeated three times) ≤19 words - Word list recall ≤75% - Mini-Mental State Examination (MMSE) ≤26/30 points | Prior dementia diagnosis  Dementia/substantial cognitive decline suspected by study physician at screening:  MMSE<20 points |
| Other factors |  | Conditions preventing cooperation  Simultaneous participation in another trial |

* CAIDE risk score described in detail in Table 2.

**Appendix Table 2.** **Cardiovascular Risk Factors, Aging, and Dementia (CAIDE) risk score**

| CAIDE Factors Points Measurements | Points | Measurement | **Total score** | **Dementia risk** |
| --- | --- | --- | --- | --- |
| **Age** |  | Population register | **0-5**  **6-7**  **8-9**  **10-11**  **12-15** | **1.0%**  **1.9%**  **4.2%**  **7.4%**  **16.4%** |
| <47 y | 0 |  |  |  |
| 47–53 y | 3 |  |  |  |
| >53 y | 4 |  |  |  |
| **Sex** |  | Population register |  |  |
| Women | 0 |  |  |  |
| Men | 1 |  |  |  |
| **Education** |  | Self-reported |  |  |
| ≥10 y | 0 |  |  |  |
| 7–9 y | 2 |  |  |  |
| 0–6 y | 3 |  |  |  |
| **Systolic blood pressure** |  | Trained study nurses measured blood pressure with a validated automatic device (Microlife WatchBP Office) with the participant in a sitting position, using the right arm, after 10 min of rest. The mean value of 2 measures was used. |  |  |
| ≤140 mm Hg | 0 |  |  |  |
| >140 mm Hg | 2 |  |  |  |
| **BMI** |  | Trained study nurses measured height (without shoes) to the nearest 0.1 cm, and weight (in light clothing). BMI was calculated by dividing the weight in kilograms by squared height in meters. |  |  |
| ≤30 kg/m2 | 0 |  |  |  |
| >30 kg/m2 | 2 |  |  |  |
| **Serum total cholesterol** |  | Fasting venous blood samples were taken, and total serum cholesterol was determined  enzymatically using commercial reagents from Abbott Laboratories on a clinical chemistry  analyzer, Architect c8000 (Abbott Laboratories, Abbott Park, IL). |  |  |
| ≤6.5 mmol/L | 0 |  |  |  |
| >6.5 mmol/L | 2 |  |  |  |
| **Physical activity** |  | Self-reported leisure-time physical activity was assessed with the question “How often do you participate in leisure-time physical activity that lasts at least 20–30 minutes and causes breathlessness and sweating?”. Response options were as follows: 1 = 5 times a week or more often; 2 = 4 times a week; 3 = 3 times a week; 4 = 2 times a week; 5 = once a week; 6 = less than once a week; 7 = I have a disability or a disease which does not enable me to exercise.  Physical inactivity was defined as frequency <2 times/wk. |  |  |
| Active | 0 |  |  |  |
| Inactive | 1 |  |  |  |

**Appendix Text 1. Description of the intervention procedures**

**All participants (control and intervention)**

Both groups had planned meetings with a study nurse at various time points (screening, baseline, and at 6 months, 12 months, and 24 months).

Both groups at the beginning of the study were provided advice on maintaining a healthy diet and engaging in physical, cognitive, and social activities that can help reduce vascular risk and prevent disability by the study nurse. At each timepoint participants underwent blood tests and had measurements taken for blood pressure, weight, body mass index, hip and waist circumference. A study physician met all the participants at screening visit and after 24 months and reviewed the medical history and performed a physical examination. All participants received the results of blood tests, accompanied by the general written information focusing on the significance of these evaluations and recommending participants to consult their primary healthcare services if they had any queries or concerns.

**Intervention group only**

*The physical exercise training programme* followed international guidelines and represented a modified version of the Dose Responses to Exercise Training (DR's EXTRA) study protocol. Training was guided by study physiotherapists at the gym and consisted of individually tailored programmes for progressive muscle strength training (1–3 times per week) and aerobic exercise (2–5 times per week), including exercises to improve postural balance. (1) Strength training and its progression were individualized by repetition maximum (RM) measurements conducted at baseline, 1, 3, 6, 9, 12, 18 and 24 months. RM was measured for all 8 muscle groups in the training protocol (knee extension and flexion, leg press, trunk rotation, upper body extension and flexion, upper arm extension and flexion). Based on RM, exercise load for the subsequent training period was set according to the load defined in the protocol (see table below).

An individual plan for aerobic training including activities preferred by each participant was first done at the time of the baseline RM measurement. Aerobic group activities were also provided. Each participant kept a diary of their daily aerobic training. At each RM measurement, the physical therapist checked the diaries together with the participant, and the plan was updated accordingly.

|  | 0-1 mo | 1-3 mo | 3-6 mo | 6-24 mo |
| --- | --- | --- | --- | --- |
| *Resistance Exercise* | | | | |
| Exercise frequency/wk | 1-2 | 1-2 | 2 | 2-3 |
| Duration of exercise, min | 30- 45 | 30- 60 | 45- 60 | 60 |
| Number of muscle groups | 8-10 | 8-10 | 8-10 | 8-10 |
| Repetitions/ set | 8-15 | 10- 20 | 8- 20 | 8- 20 |
| Load % 1RM | 40-50 | 60 | 70 | 70-80 |
| Number of sets | 2 | 2-3 | 1-3 | 2-3 |
| *Aerobic Exercise* | | | | |
| Exercise frequency/wk | 2 | 2-3 | 3-4 | 3-5 |
| Duration of exercise, min | 30-45 | 30-45 | 30-60 | 45-60 |

*The nutritional intervention* was based on the Finnish Nutrition Recommendations and was conducted by study nutritionists (3 individual sessions and 7 to 9 group sessions). Individual sessions included tailoring of the participant's diet. Group sessions provided discussions and practical exercises for facilitating lifestyle changes. Participants were advised to consume a diet with 10–20% of daily energy from proteins, 25–35% daily energy from fat (<10% from saturated plus trans fatty acids, 10–20% from monounsaturated fatty acids, and 5–10% from polyunsaturated fatty acids [including 2.5–3 g/day of omega-3 fatty acids]), 45–55% daily energy from carbohydrates (<10% from refined sugar), 25–35 g/day of dietary fibre, less than 5 g/day of salt, and less than 5% daily energy from alcohol. Energy intake facilitating 5–10% reduction in bodyweight was recommended only if necessary after taking into account BMI, health status, age, and diet of the participant. These goals were achieved by recommending high consumption of fruit and vegetables, consumption of wholegrain cereal products and low-fat milk and meat products, limiting of sucrose intake to less than 50 g/day, use of vegetable margarine and rapeseed oil instead of butter, and fish consumption at least two portions per week. (1)

*Cognitive training* included group and individual sessions. The ten group sessions were led by psychologists: six sessions with educational content on age-related cognitive changes, memory, and reasoning strategies applied to everyday activities, and four sessions for checking progress in individual computer-based training plus a visit to the local Alzheimer Association. Individual sessions consisted of computer-based training at home or at study site, conducted in two periods of six months each. Each period included 72 training sessions (three times per week, 10–15 min per session). The training programme was a web-based in-house developed computer program including several tasks adapted from protocols previously shown to be effective in shorter-term randomized controlled trials:24 executive processes (updating spatial, updating letter, updating number, and mental set shifting tasks), working memory (maintenance task), episodic memory (relational and spatial tasks), and mental speed (shape match task).(1)

*Social activities* were stimulated through the group meetings of all intervention components.(1)

*Management of metabolic and vascular risk factors* was based on national evidence-based guidelines. This involved additional meetings with the research nurse (scheduled at 3, 9, and 18 months) and the research physician (scheduled at 3, 6, and 12 months) to assess blood pressure, weight, BMI, hip and waist measurements, physical examinations, and provide guidance on lifestyle adjustments. The study physicians did not prescribe drug treatments as part of the FINGER trial; instead, they advised participants to contact their own healthcare providers or clinics if required.(1)

## **Appendix Table 3. Description of sleep, pain and depressive symptoms variables in detail.**

| **Variables** | **Assessment** |
| --- | --- |
| Sleep questions | The single questions about sleep used in this study were  1) How many hours do you sleep normally at night?  2) Do you sleep enough?  3) Have you stayed up because of worries lately?  4) Do you have trouble falling asleep without sleep medication?  5) Do you wake up early in the middle of sleep?  6) Are you more tired than others of the same age during the daytime?  7) Have you experienced insomnia or trouble sleeping during the past 30 days?  8) Have you had nightmares during the past 30 days?  Sleep quality composite index was based on 7 questions (questions 2-8 above). The answer options were on a Likert scale, and from those we rescored the answers from 0 to 100, in the same way as RAND-36 pain scale. Then the 7 questions were averaged together and 4 of the 7 questions needed to be answered in order to create the sleep quality scale. No trouble with sleeping was score 0, and 100 meaning multiple and severe sleep problems. Worse sleep quality with the sleep quality index cut-off score was set to ≥40/100, as it was estimated to reflect more severe sleep problems and sleep problems in several areas, and to characterize participants with frequent sleep problems that are either moderate or severe. |
| Bodily pain | Bodily pain was assessed using RAND-36(2)The bodily pain concept consists of two items, which describe pain severity and how much the pain disturbs daily chores at home or outside from home. The items are each scored in a Likert scale (1-5 or 1-6) and then rescored from 0 to 100 as instructed, and then averaged in the same scale together.(3) In this analysis, the scale was reversed from the original, 0 now meaning no pain, and 100 meaning the severe pain. The pain index was then divided into 2 categories. The cut-off score was calculated so that the pronounced bodily pain indicated at least average amount of pain that disturbs daily living at least some amount. Therefore, the cut-off score was set to ≥40/100. |
| Zung scale | Depressive symptoms were assessed with the Zung Self-Rating Depression Scale (4) containing 20 questions capturing the affective, psychological, and somatic symptoms associated with depression. Half of the questions were positively, and half negatively phrased. Examples included: “I feel downhearted and blue” (negative wording) and “I find it easy to make decisions” (positive wording). All questions concerned the past week, and answer options were: “A little of the time”; “Some of the time”; “Good part of the time”; and “Most of the time”. The answer options were scored from 1 to 4 or 4 to 1, depending on the phrasing. A sum score ranged from 20 to 80 points, with a higher score representing more depressive symptoms. We allowed one missing value per participant, by replacing the missing value with a question-specific average value to calculate Zung total score. |

## **Appendix Text 2. Cross-sectional results**

The associations were U-shaped for sleep duration, i.e. both short and long sleep durations were associated with more depressive symptoms and more bodily pain (p<0.001, Appendix table 3). Participants with a poor sleep quality had more depressive symptoms and pain (p<0.001), and those with depressive symptoms had more often pronounced pain (p<0.001, Appendix table 4) For sleep duration the associations were mainly U-shaped, as those sleeping 7-8 hours sleep duration had less other symptoms.

PA was not significantly associated with sleep duration of <6h (OR 1.11, 95% CI 0.55–2.25); 8–9h (OR 0.79, 95% CI 0.56–1.10); or ≥9h: (OR 0.66, 95% CI 0.40–1.10) compared with ≥7–<8h/night. There were also no associations of PA with poor sleep quality: (OR 0.83, 95% CI 0.581.18); or bodily pain: (OR 0.90, 95% CI 0.64–1.25).

| **Appendix Table 4. Baseline characteristics of FINGER participants with no physical activity information from baseline and 24month visit compared to participants with available physical activity information at both timepoints** | | | | | |
| --- | --- | --- | --- | --- | --- |
|  | **Participants with missing baseline and 24 months PA information** | | **Participants with available baseline and 24 months PA information** | |  |
|  | N |  | N |  | *p** |
| Age, mean (SD) | 159 | 69.7 (4.9) | 1100 | 68.7 (4.7) | 0.010 |
| Sex, female n (%) | 159 | 80 (50.3 %) | 1100 | 507 (46.1 %) | 0.996 |
| Education, years, mean (SD) | 158 | 9.5 (3.4) | 1099 | 10.1 | 0.046 |
| BMI, mean (SD) | 159 | 28.1 (4.4) | 1091 | 28.2 (4.8) | 0.878 |
| Poor self-rated health, n (%) | 156 | 7 (4.5 %) | 1096 | 36 (3.3 %) | 0.177 |
| Average self-rated health, n (%) |  | 66 (42 %) |  | 393 (35.9 %) |  |
| Excellent or good self-rated health, n (%) |  | 83 (53.2 %) |  | 667 (60.9 %) |  |
| Cognition (NTB total score), mean (SD) | 159 | -0.14 (0.60) | 1099 | 0.01 (0.57) | 0.002 |
| Physical activity at least 2 times a week, n (%) | 146 | 96 (65.8 %) | 1100 | 786 (71.5 %) | 0.155 |
| Sleep duration 4–6 hours per night |  | 10 (6 %) |  | 54 (5 %) | 0.055 |
| Sleep duration 6–7 hours per night |  | 38 (24 %) |  | 173 (16 %) |  |
| Sleep duration 7–8 hours per night |  | 47 (30 %) |  | 387 (35 %) |  |
| Sleep duration 8–9 hours per night |  | 46 (29 %) |  | 387 (35 %) |  |
| Sleep duration ≥ 9 hours per night |  | 16 (10 %) |  | 96 (9 %) |  |
| Sleep quality, mean (SD) | 158 | 29.4 (17.5) | 1100 | 27.8 (18.7) | 0.331 |
| Depressive symptoms, mean (SD) | 149 | 34.7 (7.8) | 1074 | 33.9 (7.4) | 0.220 |
| Bodily pain, mean (SD) | 156 | 30.2 (24.7) | 1092 | 25.1 (21.7) | 0.007 |
| At least 66% exercise adherence, N (%) | 85 | 10 (11.8 %) | 546 | 294 (53.9 %) | 0.000 |

Note. Numbers are mean, and SD unless otherwise specified

**p*-values presented in the table are t-test for normally distributed variables, Mann-Whitney test for non-normally distributed variables (Sleep quality and Bodily pain), and chi square test for categorical variables.

## **Appendix Table 5. Baseline characteristics of FINGER participants according to sleep duration.**

| **Sleep duration** | **Participants with information available, N** | **less than 6 hours**  **n=64 (5 %)** | **6 – 7 hours**  **n=211 (17%)** | **7-8 hours**  **n=434 (35 %)** | **8-9 hours**  **n= 433 (35%)** | **≥9**  **n=112 (9 %)** | **p *** |
| --- | --- | --- | --- | --- | --- | --- | --- |
| Age, years, mean (SD) | 1097 | 70.0 (4.7) | 69.3 (4.8) | 68.7 (4.6) | 68.8 (4.6) | 68.8 (5.0) | 0.167 |
| Sex, female n (%) | 1097 | 35 (55 %) | 95 (45 %) | 218 (50 %) | 186 (43 %) | 50 (45 %) | 0.154 |
| Education years, mean (SD) | 1096 | 9.3 (3.4) | 9.7 (3.4) | 10.2 (3.7) | 9.8 (3.2) | 10.5 (3.8) | 0.041 |
| BMI, mean (SD) | 1088 | 29.4 (4.9) | 28.3 (5.1) | 27.8 (4.5) | 28.1 (4.4) | 28.8 (5.5) | 0.052 |
| At least good self-reported health status | 1093 | 28 (44 %) | 100 (48 %) | 272 (63 %) | 277 (64 %) | 71 (63 %) | 0.000 |
| Cognition (NTB total score), mean (SD) | 1096 | -0.14 (0.58) | -0.08 (0.59) | 0.03 (0.58) | 0.02 (0.56) | -0.03 (0.62) | 0.061 |
| Physical activity at least 2 times a week, n (%) | 1097 | 45 (71 %) | 130 (63 %) | 321 (74 %) | 308 (72 %) | 76 (68 %) | 0.057 |
| Sleep quality, mean (SD) | 1094 | 47.4 (18.9) | 35.1 (17.7) | 24.6 (15.4) | 19.7 (13.2) | 22.7 (16.2) | 0.000 |
| Depressive symptoms, (Zung score), mean (SD) | 1071 | 38.8 (8.7) | 35.9 (7.4) | 33.3 (7.1) | 32.7 (7.0) | 35.3 (7.9) | 0.000 |
| Bodily pain, mean (SD) | 1089 | 38.0 (23.0) | 31.7 (24.1) | 23.8 (21.5) | 22.7 (19.6) | 27.3 (25.2) | 0.000 |
| Exercise adherence at least 66% in intervention group, N (%) | 628 | 13 (33 %) | 52 (51 %) | 114 (52 %) | 104 (50 %) | 19 (32 %) | 0.023 |

Note. Numbers are means (SD), unless otherwise specified.

**p*-values presented in the table are from Chi square or Fisher’s exact test, or one-way ANOVA for continuous variables and Kruskal-Wallis test for non-normally distributed variables (Sleep quality and Bodily pain).

## **Appendix Table 6. Baseline characteristics by sleep quality, pain and depressive symptoms**

|  |  | **Sleep quality** | | | **Bodily pain** | | | **Depressive symptoms** | | |
| --- | --- | --- | --- | --- | --- | --- | --- | --- | --- | --- |
|  | Participants with information available, N | Better sleep quality  N=891  (81 %) | Poor sleep quality  N= 206  (19 %) | *p** | No or little pain  N=808  (74 %) | Pronounced pain  N=281 (26%) | *p** | No or mild symptoms  N=855 (80%) | Clinically significant symptoms N=219  (20 %) | *p** |
| Age, years, mean (SD) | 1097 | 68.7 (4.7) | 69.0 (4.7) | 0.410 | 68.7 (4.7) | 68.8 (4.6) | 0.384 | 68.5 (4.6) | 69.5 (4.8) | 0.001 |
| Education years, mean (SD) | 1096 | 10.1 (3.5) | 9.8 (3.3) | 0.308 | 10.2 (3.5) | 9.7 (3.1) | 0.014 | 10.1 (3.4) | 10.1 (3.7) | 0.576 |
| BMI, mean (SD) | 1088 | 28.0 (4.6) | 29.0 (5.2) | 0.004 | 27.7 (4.4) | 29.7 (5.4) | 0.000 | 28.0 (4.6) | 28.8 (5.2) | 0.037 |
| Sleep duration | 1094 | 7.4 (0.9) | 6.7 (1.2) | 0.000 | 7.4 (1.0) | 7.2 (1.2) | 0.001 | 7.3 (1.0) | 7.2 (1.3) | 0.006 |
| Sleep quality, mean (SD) | 1097 | 19.1 (10.35) | 53.5 (11.1) | 0.000 | 22.4 (15.4) | 34.5 (18.4) | 0.000 | 22.7 (15.3) | 37.5 (18.6) | 0.000 |
| Pain, mean (SD) | 1089 | 21.8 (20.3) | 39.6 (21.8) | 0.000 | 14.6 (12.3) | 55.3 (12.8) | 0.000 | 22.4 (20.3) | 35.9 (24.1) | 0.000 |
| Depressive symptoms, mean (SD) | 1085 | 32.5 (6.7) | 39.8 (7.3) | 0.000 | 32.5 (6.8) | 37.9 (7.6) | 0.000 | 31.1 (4.9) | 45.0 (4.8) | 0.000 |
| Cognition (NTB total score), mean (SD) | 1096 | 0.02 (0.57) | -0.02 (0.56) | 0.395 | 0.05 (0.57) | -0.95 (0.55) | 0.000 | 0.06 (0.57) | - 0.13 (0.56) | 0.000 |
| Self-reported current health status, at least good. N (%) | 1093 | 589 (66.4 %) | 77 (37.4 %) | 0.000 | 562 (69.7 %) | 99 (35.1 %) | 0.000 | 574 (67.4 %) | 78 (35.6 %) | 0.000 |
| Exercise adherence in intervention group, at least 66%. N (%) | 545 | 236 (54.1 %) | 57 (52.3 %) | 0.731 | 226 (56.1 %) | 66 (47.5 %) | 0.080 | 236 (56.0 %) | 48 (44.9 %) | 0.038 |

Note. Numbers are mean, and SD unless otherwise specified

**p*-values presented in the table are t-test for normally distributed variables, Mann-Whitney test for non-normally distributed variables (Sleep quality and Bodily pain), and chi square test for categorical variables.

## **References**

1. Ngandu T, Lehtisalo J, Solomon A, et al. A 2 year multidomain intervention of diet, exercise, cognitive training, and vascular risk monitoring versus control to prevent cognitive decline in at-risk elderly people (FINGER): a randomised controlled trial. *Lancet*. 2015;385(9984):2255-2263. doi:10.1016/S0140-6736(15)60461-5

2. Hays RD, Morales LS. The RAND-36 measure of health-related quality of life. *Annals of Medicine*. 2001;33(5):350-357. doi:10.3109/07853890109002089

3. Aalto AM, Aro AR, Teperi J. RAND-36 TERVEYTEEN LIITTYVÄN ELÄMÄNLAADUN MITTARINA.

4. Zung WW. From art to science. The diagnosis and treatment of depression. *Archives of General Psychiatry*. 1973;29(3):328-337. doi:10.1001/archpsyc.1973.04200030026004 [doi]
